# The Heart Beats as It Breathes, or Is It the Other Way Around?

**DOI:** 10.3390/medicina59081431

**Published:** 2023-08-08

**Authors:** Nicola Vitulano

**Affiliations:** Cardiology and Coronary Intensive Care Unit, Ospedale Generale F. Miulli, Strada Provinciale per Santeramo km 4,100, Acquaviva delle Fonti(BA), 70021 Bari, Italy; nicola.vitulano@gmail.com

One third of human life is spent sleeping, thus the importance of sleep in the maintenance of correct homeostatic balance is well established. During the night, restoring changes go on and psychophysical capacities are reduced. One hundred years have passed since the first article linking heart activity and sleep [1] and currently the relationship between cardiac disease and sleep respiratory disturbances is closely studied.

Sleep-related breathing disorders are frequently present in patients with hypertension, ischemic heart disease, heart failure and arrhythmias. Perhaps in the setting of cardiopulmonary relationship, hypertension was one of the first aspects detected when it was found that nocturnal hypoxaemia played a key-role in the aetiology of systemic hypertension in patients with sleep-related breathing disorders [2]. Instead, the last article in the field of ischemic heart disease points out that individuals at established or at high risk of obstructive sleep apnoea have a higher likelihood of the presence of coronary plaque [3]. Screening for sleep apnoea in patients with exacerbated heart failure is necessary to detect this important comorbidity in the complex setting of cardiac pump failure because of the beneficial effects of its treatment among the therapeutic options for these kinds of patients. In the context of cardiac arrhythmias there was probably the greatest interest about the relationship between nightly apnoeic events and heart rhythm. Indeed, patients affected by obstructive sleep apnoea show altered inflammatory pathways, and these triggers could be at the root of the development of arrhythmias. The pathophysiological mechanisms of atrial fibrillation (AF) in patients with obstructive sleep apnoea are detected by using atrial tissue samples and assessment of several biomarkers. It is found that dysregulated miRNA, interstitial fibrosis and Connexins expression create the substrates of electro-anatomical disarrangement linked to higher risk of AF [4], the most common arrhythmias affecting cardiac patients above all over the age of fifty. Several aspects need to be considered in the pathogenesis of AF onset and its maintenance, traceable not only to atrial fibres disarray, cardiovascular risk factor (diabetes, atherosclerosis, chronic renal failure) or extracardiac noxa such as nightly hypoxemia. Neurovegetative system is essential to control normal cardiac physiology, mechanical contraction and at the same time pulmonary functions; it could be in the middle of these two systems such as a trait d’union between altered cardiac rhythm and nightly dysregulation of oxygen flow.

In recent years, a contribution of the autonomic nervous system (ANS) is highlighted for a better comprehension of several mechanisms involved in the heart–sleep–breath interaction. Alterations in sympathetic tone during apnoeic events stimulate sympathetic activation such as severe intermittent hypoxemia, acidosis, and hypercapnia can result in sympathetic activation leading to heart rate elevation. Thus, the link between obstructive sleep apnoea and arrhythmias, above all AF, may be a combination of sympathetic and parasympathetic drivers and electrophysiologic substrate modification. At the same time initiation and maintenance of AF may be partially influenced by intrinsic cardiac ANS.

In the ANS, fibres travel from the central nervous system to the ganglia of visceral effectors organs. Ganglia of the parasympathetic division of the heart are distributed in mostly in epicardial regions, identified in humans in the posterior-superior surface of the right atrium adjacent to the superior vena cava, junction and right atrium; the posterior-superior surface of the left atrium; the interatrial groove; the posterior-medial surface of the left atrium; the posterolateral surface of the left atrium; and the ligament of Marshall densely innervated by the left vagus nerve (Figure 1).

Most of the mentioned localizations are the potential triggers during catheter ablation of AF and subsequent potential applications for neuromodulation in appropriately selected patients is the present and the future in the electrophysiologic study of the atrial activity and AF. Following the discovery that ganglia stimulation may result in triggered activity in PVs and fractionated EGMs sites thought to maintain AF, modulating or eliminating key neural connections to the heart by using endocardial ablation could be a target that is reflected also in global circadian activity, not only about the heart. A stable heart rhythm could have a positive influence on nightly desaturation and sleep patterns [5]. Heart rate stability produces a more regular blood flow, resulting in a different receptors activation for the breathing control at various levels. In fact, AF is a period of high and irregular heart rate, with consequent irregular blood distribution to periferical and central control system. Thus, regular rhythm or the electrophysiological substrate modification remodelling could play a pivotal role in the restoration of regular blood circulation; autonomic balance through ablation of ganglionated plexi might have a positive impact for a regular perfusion of the pulmonary circulation with a consequent decrease in pulmonary receptors activation. Constant glomus cells surveillance monitors positive stabilization in PO2 and PCO2 values producing biochemical information sent to central control with the aim to stabilize nightly respiratory pattern. Thus, the ANS may be in the centre of this big vicious loop involving heart rate and cardiac activity, atrial arrhythmic burden, nightly apnoeic events with following hypoxemic trigger activities and their impact on cardiac performance (Figure 2).

If there are nightly respiratory disorders, the cardiac work is energy-consuming; but probably at the same time a heart that beats as a metronome gives the pace for a regular and restoring nightly breathing. It is clear that disturbed sleep, due to disturbed nightly breath, is not healthy for the heart.

## Figures and Tables

**Figure 1:**
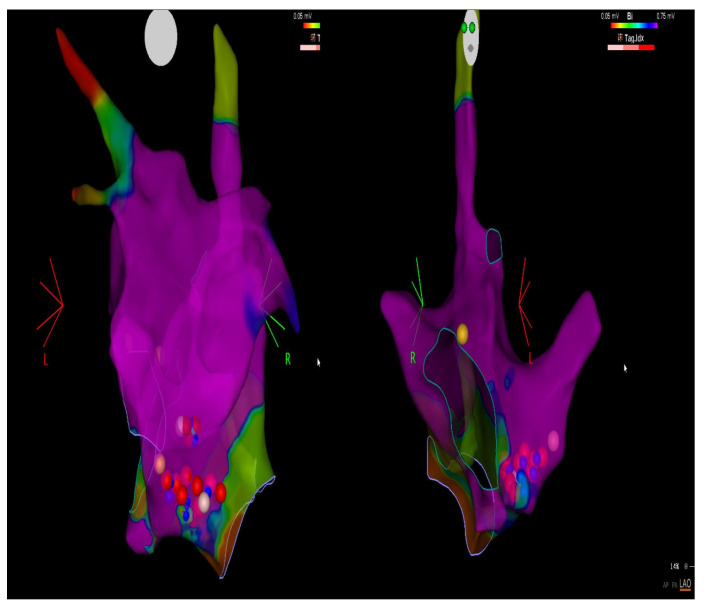
CARTO-map image: an electro-anatomical recreation of the right atrium and interatrial septum, the violet part is the electrically normal tissue; the yellow, blue and white dots are the positions of the ganglia; the red ones are the triggers for radiofrequency application for neuromodulation.

**Figure 2:**
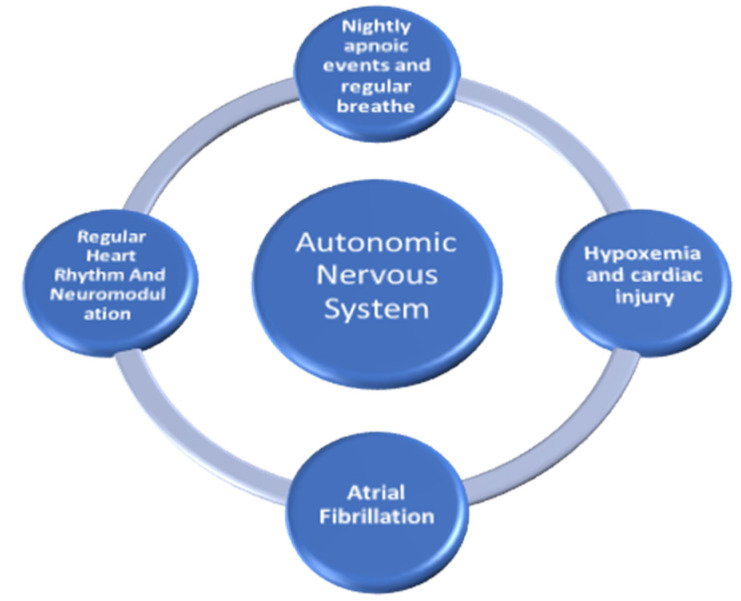
The autonomic nervous system may represent the joining link between atrial arrhythmias and nightly apnoic events causing hypoxemia.
